# Pro-Osteogenic Effect of the Nutraceutical BlastiMin Complex^®^ in Women with Osteoporosis or Osteopenia: An Open Intervention Clinical Trial

**DOI:** 10.3390/ijms25168565

**Published:** 2024-08-06

**Authors:** Sofia Sabatelli, Emanuele-Salvatore Scarpa, Angelica Giuliani, Chiara Giordani, Jacopo Sabbatinelli, Maria Rita Rippo, Sara Cabodi, Barbara Petrini, Giancarlo Balercia, Gilberta Giacchetti

**Affiliations:** 1 Clinic of Endocrinology and Metabolic Diseases, Department of Clinical and Molecular Sciences, Università Politecnica delle Marche, 60126 Ancona, Italy; s.sabatelli@pm.univpm.it; 2 R&D Department, Mivell S.r.l.s., 61032 Fano, Italy; e.scarpa@mivell.com; 3 Department of Clinical and Molecular Sciences (DISCLIMO), Università Politecnica delle Marche, 60126 Ancona, Italy; angelica.giuliani@staff.univpm.it (A.G.); j.sabbatinelli@staff.univpm.it (J.S.); m.r.rippo@staff.univpm.it (M.R.R.); 4 Clinic of Laboratory and Precision Medicine, IRCCS Istituto Nazionale di Ricovero e Cura per Anziani (INRCA), 60126 Ancona, Italy; 5 R&D Department, Diatech Pharmacogenetics S.r.l., 60035 Jesi, Italy; sara.cabodi@diatechpharmacogenetics.com (S.C.); barbara.petrini@diatechpharmacogenetics.com (B.P.); 6 Division of Endocrinology, Department of Clinical and Molecular Sciences, Università Politecnica delle Marche, 60126 Ancona, Italy; giancarlo.balercia@ospedaliriuniti.marche.it

**Keywords:** osteoporosis, nutraceuticals, osteoblasts, P1NP, CTX, BMD, CRP

## Abstract

Osteoporosis is a chronic disease that affects millions of patients worldwide and is characterized by low bone mineral density (BMD) and increased risk of fractures. Notably, natural molecules can increase BMD and exert pro-osteogenic effects. Noteworthily, the nutraceutical BlastiMin Complex^®^ (Mivell, Italy, European Patent Application EP4205733A1) can induce differentiation of human bone marrow mesenchymal stem cells (BM-MSCs) in osteoblasts and can exert in vitro pro-osteogenic and anti-inflammatory effects. Thus, the purpose of this study was to verify the effects of BlastiMin Complex^®^ on bone turnover markers (BTMs) and BMD in patients with senile and postmenopausal osteopenia or osteoporosis. The efficacy of BlastiMin Complex^®^ on BTMs in serum was evaluated through biochemical assays. BMD values were analyzed by dual-energy X-ray absorptiometry (DXA) and Radiofrequency Echographic Multi Spectrometry (R.E.M.S.) techniques, and the SNPs with a role in osteoporosis development were evaluated by PCR. Clinical data obtained after 12 months of treatment showed an increase in bone turnover index, a decrease in C-reactive protein levels, and a remarkable increase in P1NP levels, indicating the induction of osteoblast proliferation and activity in the cohort of 100% female patients recruited for the study. These findings show that the nutraceutical BlastiMin Complex^®^ could be used as an adjuvant in combination with synthetic drugs for the treatment of osteoporosis pathology.

## 1. Introduction

Osteoporosis is a bone disorder in which the balance between bone resorption and bone formation is disrupted, resulting in an increase in bone resorption that decreases bone mineral density (BMD) [1]. The World Health Organization (WHO) defines osteoporosis as a “progressive systemic skeletal disease characterized by low bone mass and microarchitectural deterioration of bone tissue, with a consequent increase in bone fragility and susceptibility to fracture” [2]. Osteoporosis is a common disease, affecting more than 200 million patients worldwide [3]. The development of this pathology is influenced by several factors, including age and sex, and is divided accordingly into primary and secondary osteoporosis. Noteworthily, bone aging is characterized by an imbalance in the physiological and pathological processes of osteogenesis and osteoclastogenesis, resulting in remarkable bone loss and the presence of a particular inflammation state indicated by term inflammaging, leading to the development of several age-related bone diseases, including osteoporosis [4]. Notably, primary osteoporosis includes postmenopausal osteoporosis, which is characterized by a decreased production of estrogen, determining bone loss. On the other hand, secondary osteoporosis is caused by disease or drug exposure and is characterized by reductions in bone function, often due to glucocorticoid use, hyperparathyroidism, hypogonadism, excessive alcohol consumption, or poor nutrition [5,6,7,8,9].

Noteworthily, osteoporosis is a disease that has no major symptoms unless a fracture occurs [10]. BMD T-scores are used for diagnosis, where T-scores > −1.0 standard deviation (SD) denote normal bone mass; T-scores between −1.0 and −2.5 SD are defined as osteopenia; T-scores < −2.5 SD are indicative of osteoporosis [11,12]. Current osteoporosis treatments are either anti-resorptive, bone-forming, or dual-acting (including both types of treatment) [13]. Anti-resorptive drugs include bisphosphonates (alendronate, clodronate), the anti-receptor activator of NF-kB ligand (*RANKL*) antibody (Denosumab), and selective estrogen receptor modulators (SERMs) [13,14]. Bone-forming drugs include teriparatide and the antibody Romosozumab, which induce an increase in osteoblast proliferation and activity [13,14,15]. Interestingly, it was shown that the nutraceutical BlastiMin Complex^®^ (Mivell, Italy) can induce the differentiation of human BM-MSCs in osteoblasts. Noteworthily, the pro-mineralizing component (orthosilicic acid and vitamin K2) of this nutraceutical, in combination with the anti-inflammatory component of the formulation (curcuminoids, polydatin, and quercetin), acts synergistically in inducing the expression of the osteogenic marker *COL1A1* in young BM-MSC cells [16]. Furthermore, the combination of these five bioactive compounds can synergistically increase the expression levels of the osteogenic marker *ALP* in senescent human BM-MSCs and decrease the protein levels of p-p38, phospho-nuclear factor kappa B (p-NF-kB), monocyte chemoattractant protein-1 (MCP-1), and interleukin-8 (IL-8), demonstrating the pro-mineralizing and anti-inflammatory effects of the nutraceutical BlastiMin Complex^®^ [17]. This scientific evidence indicates that the natural molecules of BlastiMin Complex^®^ can inhibit the Senescence Associated Secretory Phenotype (SASP) of senescent BM-MSCs, counteracting the ability of these cells to release those cytokines and interleukins that can transform the young BM-MSCs into senescent BM-MSCs. The biological effects exerted by this nutraceutical avoid the depletion of the cell population of young BM-MSCs, which can differentiate into new osteoblasts and then induce the generation of new bone tissue [17].

Bone mass is maintained by continuous bone remodeling through bone formation by osteoblasts and bone resorption by osteoclasts [18,19]. Osteoclasts differentiate from hematopoietic stem cells and are activated by macrophage colony-stimulating factor and *RANKL* to attach to bone and begin resorption [20,21]. Activated osteoclasts induce bone resorption through bone mineral dissolution and bone degradation via proteolytic enzymes and hydrochloric acid secretion [22,23]. The main proteolytic enzymes released from osteoclasts are cathepsin K and matrix metalloproteinase-9 (MMP-9) [24]. Furthermore, the *RANKL* interaction with its receptor *RANK* activates additional signaling pathways that lead to an increase in osteoclast activity [3]. On the other hand, osteoblasts differentiate from bone marrow mesenchymal stem cells (BM-MSCs), produce hydroxyapatite, and secrete type I collagen (codified by Collagen Type I Alpha 1 Chain (*COL1A1*)), osteocalcin (OCN), and bone alkaline phosphatase (bALP), enabling the formation of new bone tissue [25,26,27]. Noteworthily, OCN is a calcium-binding peptide secreted by mature osteoblasts and is the most abundant non-collagen protein found in bones. Most OCN secreted by osteoblasts is incorporated into the organic matrix that will later ossify into bones; however, a fraction of this protein is secreted into the circulation and can be identified as a bone formation marker, indicating the activity levels of osteoblasts [27]. Notably, osteoblast proliferation is regulated by several signaling molecules, including Runt-related transcription factor 2 (Runx2) and osterix [28]. In particular, Runx2 levels are increased by bone morphogenetic proteins (BMPs), Wnt/β-catenin levels, and receptors for lipoprotein receptor-related proteins 5 and 6 (LRP5/6). Noteworthily, osteoblasts promote bone formation by synthesizing an extracellular matrix to maintain bone mass [29].

As osteoporosis emerges directly from a decrease in bone mass and alterations in the number or activities of osteoblasts and osteoclasts, it follows that biomarkers of the activity of these cells reflect current levels of bone turnover and are identified in the clinic as bone turnover markers (BTMs), which can be categorized as reflecting either bone resorption or formation [27]. Osteoblasts secrete type I collagen as an intact molecule containing the N- and C-terminal propeptides, which are subsequently cleaved in the extracellular space. Thus, N- and C-terminal propeptides of type I collagen (P1NP and PICP) levels are markers of type I collagen secretion by osteoblasts. Notably, P1NP is produced by the enzyme procollagen-N-endopeptidase of the osteoblasts after the cleavage of the protein type I collagen, and the increase in this BTM indicates an induction of osteoblast activity and the formation of new bone tissue [27]. Noteworthily, C- and N-terminal telopeptides of type I collagen (CTX and NTX) are both fragments of type I collagen obtained from the telopeptide region. The telopeptides are cleaved during osteoclastic resorption of bone, resulting in their liberation into the circulation at a rate proportional to bone resorption activity; in particular, CTX is the specific product of cathepsin K-mediated cleavage of type I collagen exerted by osteoclasts [27]. Osteoclasts represent the main target of current drug therapy for osteoporosis, but there is an ever-increasing number of therapeutic approaches that target osteoblasts, including natural molecules [3].

The purpose of this study was to verify the effects of the nutraceutical BlastiMin Complex^®^ on BTMs (such as P1NP), bone density, and quality in women with senile and postmenopausal osteopenia or osteoporosis. The evaluation of the BlastiMin Complex^®^-mediated modulation of the levels of the osteoblasts-related biomarker P1NP is associated with the idea of an innovative therapeutic approach for osteoporosis based on nutraceuticals: increasing the activity and proliferation of osteoblasts and avoiding focusing solely on the inhibition of osteoclast activity.

## 2. Results

### 2.1. Effects of BlastiMin Complex^®^ on Serum BTMs, CRP, and BMD Values

We evaluated the effects of the nutraceutical BlastiMin Complex^®^ on the serum biomarkers of osteogenesis, the marker of systemic inflammation CRP, and the BMD values (Table 1).

These results were obtained after both 6 months and 12 months of the BlastiMin Complex^®^ treatment administered to the forty-two patients that completed the 12-month treatment since two patients withdrew from the study because of discontinued intervention. The results indicate that there was not a variation of the vitamin D2 + D3 levels in the serum, while there was a statistically significant increase after the 6-month treatment of the serum levels of the pro-osteogenic marker P1NP (*p* = 0.014), which suggests an increase in the number and activity of osteoblasts [27]. Noteworthily, after 12 months of BlastiMin Complex^®^ treatment, there was a further significant increase in P1NP levels (*p* < 0.001) (Table 1). Notably, the clinical data also show a significant increase, after 6 months of treatment, in the serum levels of CTX (*p* = 0.017), which derives from the cathepsin K-mediated cleavage of the Type I Procollagen [27]. Noteworthily, after 12 months of BlastiMin Complex^®^ treatment, the serum levels of the bone resorption marker CTX were not significantly different when compared with the t_0 months_ values (*p* = 0.085). Moreover, our results indicate that there was a slight increase in the bone turnover index after 12 months of treatment (Table 1). In addition, the clinical data show that there was no modulation of the levels of the markers OCN and bALP after 6 months and 12 months of nutraceutical treatment. On the contrary, there was a significant decrease in the serum levels of the pro-inflammatory marker CRP after 12 months of treatment (*p* < 0.001) in a subpopulation of 20 of the 42 patients, whose CRP levels at t_12 months_ were not below the detection limit (0.03 mg/dL) of the kit used to evaluate the serum CRP levels (Table 1). Furthermore, there was no variation of both the femoral and lumbosacral BMD of the 42 patients after both 6 and 12 months of BlastiMin Complex^®^ treatment (Table 1). Interestingly, the clinical data indicated a value of L1–L4 T-score of −1.512 ± 1.029 and −1.551 ± 1.014 at t_0 months_ and t_12 months_, respectively, and a value of femoral neck T-score of −1.514 ± 0.607 and −1.515 ± 0.687 at t_0 months_ and t_12 months_, respectively (Table 2).

In addition, the non-worsening of the BMD values after 12 months of treatment was confirmed using the REMS technique, which indicated a value of −1.96 ± 0.68 at t_0 months_ and −2.00 ± 0.71 at t_12 months_ for the lumbosacral region and a value of −1.88 ± 0.74 at t_0 months_ and −1.98 ± 0.74 at t_12 months_ for the femoral region (Table 2).

### 2.2. Analysis of Selected SNPs of COL1A1, RANKL, RANK, VDR, and CTR Genes

The DNAs obtained from the blood samples of the enrolled patients were used as templates to analyze the genotypes of the patients, focusing on selected SNPs that play a role in the development and worsening of osteoporosis pathology; the results are reported in Table 3. Notably, 27.3% of patients are in a condition of mutant homozygosity for the SNP of the *RANKL* gene (rs9594759 C>T), and 18.2% of patients are in a condition of mutant homozygosity for another SNP of the *RANKL* gene (rs9594738 C>T). Furthermore, 25% of patients are in a condition of mutant homozygosity for an SNP of the Vitamin D Receptor (*VDR*) gene (rs17879735 G>T). This mutation is associated with a reduced BMD and increased risk of fractures [30]. Noteworthily, the percentage of homozygous mutant patients for the two *RANKL* SNPs rs9594759 C>T and rs9594738 C>T are very similar to the percentage of the homozygous mutant individuals of the Italian population (29.0% and 18.6%, respectively), as reported [31,32], while the percentage of the homozygous mutant patients for the *VDR* SNP rs17879735 G>T is higher when compared to the corresponding percentage of homozygous mutant individuals of the Italian population (18.5%), as reported [33]. Interestingly, 4.5% of the patients were in a condition of homozygous mutant for the SNP of the *COL1A1* gene (rs1800012 G>T), and 2.3% of patients were in a condition of homozygous mutant for the SNP of the *CTR* gene (rs1801197 T>C). Notably, a relationship between the SNP rs1800012 G>T of *COL1A1* and significantly decreased FN and LS BMD values in osteoporotic postmenopausal women was reported [34]. Furthermore, our results indicate that 6.8% of the patients were in a condition of homozygous mutant for the SNP of the *RANK* gene (rs3018362 A>G) (Table 3).

Noteworthily, the analysis of the clinical data of a subpopulation of 12 patients (among the cohort of 42 patients that completed the study) who are homozygous mutants for the SNP of the *RANKL* gene (rs9594759 C>T) associated with an increase in osteoclast activity [35] indicated that there was a significant increase in CTX levels after 6 months of treatment (414.16 ± 111.21 pg/mL at t_6 months_ vs. 318.36 ± 88.11 pg/mL at t_0 months_, * *p* = 0.038) in this subpopulation as well. Furthermore, the serum CTX levels after 12 months of nutraceutical treatment were not significantly different when compared with the baseline values (359.07 ± 117.80 pg/mL at t_12 months_ vs. 318.36 ± 88.11 pg/mL at t_0 months_, *p* = 0.361). These results indicated that the treatment response of this subpopulation was similar to the response of the entire cohort of patients, even if the genotype of these 12 patients was correlated to an increase of +30.10% of serum CTX levels at t_6 months_ (+12.90% CTX levels at t_6 months_ considering the entire cohort of patients) and an increase of +12.80% of serum CTX levels at t_12 months_ (+6.50% CTX levels at t_12 months_ considering the entire cohort of patients).

## 3. Discussion

Osteoporosis is a chronic disease that affects millions of patients worldwide and is most common in menopausal women. The main characteristics of osteoporosis are low BMD, increased risk of fractures due to deterioration of the bone architecture, decrease in BTMs associated with osteoblasts, and increase in BTMs associated with osteoclasts [3]. BTMs are a series of protein or protein derivative biomarkers released during bone remodeling by osteoblasts or osteoclasts, and they can offer prognostic information on fracture risk that supplements radiographic measures of bone mass. BTMs respond rapidly to changes in bone physiology; therefore, they have utility in determining patient response to and compliance with therapies for osteoporosis based on several drugs [27]. Notably, increasing benefits of natural remedies for bone health provide an alternative strategy for treating osteopenia or osteoporosis [36]. In fact, it was reported that nutritional phytochemicals present in fruits, vegetables, and cereals can increase BMD through several molecular mechanisms, in addition to having beneficial effects on bone homeostasis and health, exerted through the decrease in bone resorption and the enhancement of osteoblastic proliferation and activity [17,36].

Noteworthily, our results show that after the treatment with the nutraceutical BlastiMin Complex^®^, there was a statistically significant increase in P1NP levels in the serum of the patients after both 6 and 12 months of treatment, suggesting that this nutraceutical induced an increase in osteoblast proliferation and activity since P1NP is the specific BTM used in clinical trials to evaluate the effects of the investigated treatments towards the osteoblast activity [27]. In fact, P1NP is a peptide that derives from the cleavage of the Type I Procollagen protein mediated by the catalytic activity of Pro-Collagen N-endopeptidase, an enzyme that can be found in osteoblasts but not in osteoclasts [27].

Notably, it was reported that the increase in osteoblast proliferation leads to an increase in *RANKL* levels, a signaling molecule produced by osteoblasts to recruit osteoclasts and increase their activity to perform the remodeling process of the new bone tissue [37]. The interaction of *RANKL* with its receptor *RANK* and the consequent induction of osteoclast activity could explain the increased CTX values observed after 6 months of BlastiMin Complex^®^ treatment in the serum of the patients [38,39]. Interestingly, our results show that there was a further increase in the P1NP levels after 12 months of BlastiMin Complex^®^ treatment, and we hypothesize that these data could be explained by the osteoclast-induced EphrinB2 (EFNB2)-Ephrin type-B receptor 4 (EphB4) interaction, as suggested by other authors [37]. Notably, it was reported that EFNB2 is a protein expressed in osteoclasts and released by these cells, while its receptor EphB4 is expressed in osteoblasts, and the interaction of the ligand EFNB2 with its receptor EphB4 leads to an increase in osteoblast proliferation and activity [37], but additional experiments need to be performed in the future to confirm this hypothesis.

Notably, after 12 months of the nutraceutical treatment, there was only a slight and non-significant increase in the serum CTX levels when compared with the values of this BTM obtained at t_0 months_. Since it was shown that the combination of the natural molecules of BlastiMin Complex^®^ can reduce the protein levels of the pro-inflammatory transcription factor p-NF-kB in human BM-MSCs [17], and that p-NF-kB can increase the levels of *RANKL* and, consequently, the activity of osteoclasts through the molecular pathway p-NF-kB/*RANKL*/*RANK* [40], we hypothesize that the lack of a further increase in CTX levels after 12 months of nutraceutical treatment could be determined by the BlastiMin Complex^®^-mediated decrease in p-NF-kB levels in the osteoblasts of the patients, but additional experiments should be performed in the future to deeply investigate the specific molecular pathways modulated by the nutraceutical formulation of BlastiMin Complex^®^ in these patients. Noteworthily, the data obtained in the subpopulation of 12 patients homozygous mutant for the SNP rs9594759 C>T of the *RANKL* gene indicated a higher 6-month increase in the serum CTX levels when compared with the 6-month increase in CTX values obtained in the cohort of 42 patients, but these results should be carefully evaluated because the number of patients of the subpopulation is too small to draw any remarkable conclusion. In fact, the future perspective is to carry out a clinical study with a higher number of patients to establish the effect of specific SNPs on the response of enrolled patients to the treatment with BlastiMin Complex^®^. Interestingly, our clinical data also indicate that after 12 months of BlastiMin Complex^®^ treatment, there was a slight increase in the BTI, which is a clinical parameter commonly used in clinical practice to evaluate the formation of new bone tissue [41]. Notably, our results show that there was no modification of the vitamin D2 + D3 levels in the serum of the patients after both 6 and 12 months of BlastiMin Complex^®^ treatment. Calcium and vitamin D3 intake determine an increase in the inorganic matrix of the bone, but the necessity to increase the organic matrix of bone by enhancing collagen levels should also be considered [27]. Both the in vitro data showing a synergistic and significant BlastiMin Complex^®^-mediated increase in *COL1A1* (the gene that codifies Type I Procollagen) levels [16] and the clinical data reporting a significant increase in the P1NP marker in the serum of the 42 patients suggest that the treatment with the nutraceutical BlastiMin Complex^®^ induces an increase in the proliferation and activity of the osteoblasts, which can secrete collagen [27]. The increase in the osteoblasts’ number can then lead to the augmentation of collagen levels and, consequently, of the organic matrix of bones. These biological effects could determine the formation of new bone tissue and counteract the detrimental effects of osteoporosis pathology. Noteworthily, it was reported that the pro-inflammatory marker CRP plays a role in increasing the risk of osteoporotic fracture in patients [42]. Our results show that there was a decrease in CRP protein levels after BlastiMin Complex^®^ treatment at t_12 months_, indicating that the therapeutic approach based on the formulation of this nutraceutical can improve this clinical parameter. Since it was shown that the levels of cathepsin K (the enzyme which produces CTX) in the serum of the patients were positively correlated with CRP levels [43], and our results indicate that after 12 months of the BlastiMin Complex^®^ treatment, there was a decrease in the serum levels of CTX, when compared with the values of this BTM obtained at t_6 months_, we hypothesize that the statistically significant decrease in serum CRP levels obtained only after 12 months of treatment could be associated with the BlastiMin Complex^®^-mediated inhibition of the NF-kB/*RANKL*/*RANK*/cathepsin K pathway [40], but additional experiments should be performed in the future to investigate the molecular markers that led to a decrease in serum CRP levels in our study.

Notably, it was also shown that individual natural molecules can have pro-osteogenic effects and play a role in the improvement of bone health, as it was reported for resveratrol [44,45]. The Resveratrol for Healthy Aging in Women (RESHAW) trial is a 24-month randomized, double-blind, placebo-controlled, two-period crossover intervention conducted to evaluate the effects of resveratrol (75 mg twice daily) on bone health and well-being in postmenopausal women [45]. The clinical data indicate that the resveratrol treatment improved the LS and FN BMD values and led to a reduction in the levels of the bone resorption marker CTX, but this polyphenol was not able to increase P1NP levels in the patients [45]. Notably, interindividual variability in therapy response represents one of the most relevant issues for the correct management of patients in clinical practice. The scientific evidence of recent years indicates how this aspect can also be relevant for the individual response to treatments with drugs or nutraceuticals. Interestingly, it was reported that hereditary factors are an important component of the diversity in individual response to both synthetic drugs and nutraceuticals, thus attributing to genes’ SNPs the ability to influence the variability of response to any active ingredient used for therapeutic approaches in particular when the treated patients are in a condition of mutant homozygosity for specific genes associated with the development of specific pathological conditions [46]. Noteworthily, the analysis of the SNPs of the enrolled patients indicated that 27.3% of patients are homozygous mutants for the SNP of the *RANKL* gene (rs9594759 C>T) and that 18.2% of patients are homozygous mutants for another SNP of the *RANKL* gene (rs9594738 C>T). These mutations are associated with an augmentation of the protein levels of *RANKL*, the ligand that interacts with *RANK* and induces an increase in the activity of osteoclasts, resulting in a worsening of the osteoporosis condition of the patients [35]. Notably, among the five investigated SNPs for the *VDR* gene, the rs17879735 G>T polymorphism is the most interesting one; in fact, 25% of patients are homozygous mutants for this SNP, and this mutation is associated with a reduction in BMD values and increased risk of fractures in osteoporotic patients [30]. In addition, even if only 4.5% of the patients were in a condition of homozygous mutant for the SNP of the *COL1A1* gene (rs1800012 G>T), the role of this polymorphism should not be underestimated because a relationship between this mutation and significantly decreased LS and FN BMD values in the postmenopausal population of osteoporotic patients was reported in a recent study [34]. Noteworthily, 6.8% of the patients were in a condition of homozygous mutant for the SNP of the *RANK* gene (rs3018362 A>G), which is associated with osteoporosis development of the lumbar spine [47]. Notably, it was shown that the homozygous mutant genotype for the SNP rs1800012 G>T of the *CTR* gene represents a less favorable genotype in terms of BMD values and leads to osteoporosis development [48].

Regarding the limitations and strengths of our study, potential limitations of the present study include the absence of a control (placebo) group, while its strengths include treatment adherence, the increase in the pro-osteogenic marker P1NP, and the specific induction of the osteoblast activity mediated by the nutraceutical BlastiMin Complex^®^, while most of the current drugs (like bisphosphonates and the antibody Denosumab) used in therapies for osteoporosis target the osteoclasts, leading to the reduction in the activity of these cells. Furthermore, another strength of this study is represented by the analysis of the patient’s SNPs with a role in osteoporosis development. These genetic polymorphisms should be considered in the development of innovative therapeutic approaches for personalized medicine to improve the therapies for osteoporosis pathology.

## 4. Materials and Methods

### 4.1. Study Design

We carried out an open-label and single-arm intervention study to evaluate the pro-osteogenic efficacy of the nutraceutical BlastiMin Complex^®^ in 44 patients with bone densitometry examination T-score > −3 and <−1 and, therefore, not eligible in the first instance for a therapeutic clinical approach with the administration of oral drugs but eligible for interventions on lifestyle, nutrition, and the introduction of nutraceuticals. As 2 patients withdrew from the study, 42 patients completed the 12 months of treatment with the nutraceutical BlastiMin Complex^®^. Since our study is an open-label and single-arm intervention study, the only applicable type of bias is represented by the reporting bias, as described by Mansournia, M.A. et al. [49]. As the primary endpoint of this clinical trial was the non-worsening of the T-score values, we decided to use two different techniques (DXA and REMS) to ensure that the T-score values did not worsen after 12 months of nutraceutical treatment, and we reported the obtained data in the results section. Furthermore, we discussed both the statistically significant and the non-statistically significant results obtained for the investigated serum biomarkers after 6 months and 12 months of BlastiMin Complex^®^ treatment.

The study was conducted in accordance with the Declaration of Helsinki; the protocol of the clinical trial was approved by the Ethical Committee of “Università Politecnica delle Marche”, and before the inclusion in the study of the patients, their informed consent was obtained. Inclusion criteria were as follows: ≥50 years and ≤75 years; subjects of both sexes; T-score between minus 3.0 and minus 1.0; postmenopausal and senile forms of osteoporosis; serum levels of 25-hydroxyvitamin D not less than 20 ng/mL. Moreover, exclusion criteria were as follows: inability, impossibility, or unwillingness to sign the written consent; forms of secondary osteoporosis caused by other pathological conditions, including prolonged immobilization or drug use according to the “Società Italiana dell’Osteoporosi, del Metabolismo Minerale e delle Malattie dello Scheletro” (SIOMMMS) guidelines; therapy with oral anticoagulants; endocrine diseases (hypothyroidism, hyperthyroidism, insulin-dependent diabetes or diabetes associated with macrovascular complications); ongoing acute illness; ongoing cancer or life expectancy <1 year; allergy/intolerance to one or more components of the treatments; participation in another clinical intervention study in the previous three months; presence of cognitive disorders and other impediments that do not guarantee correct adherence to the study treatments; abnormal liver or kidney function; consumption of more than 2 alcohol units per day (one alcohol unit corresponds to 12 g of ethanol); intake of bisphosphonate drugs or drugs related to the treatment of osteoporosis in the previous 6 months; estrogen therapy in the previous 6 months; intake of fluorides for more than 3 months in the previous 2 years; intake of systemic glucocorticoids for more than a month in the last year; intake of antiepileptics.

### 4.2. Treatment

**Figure 1:**
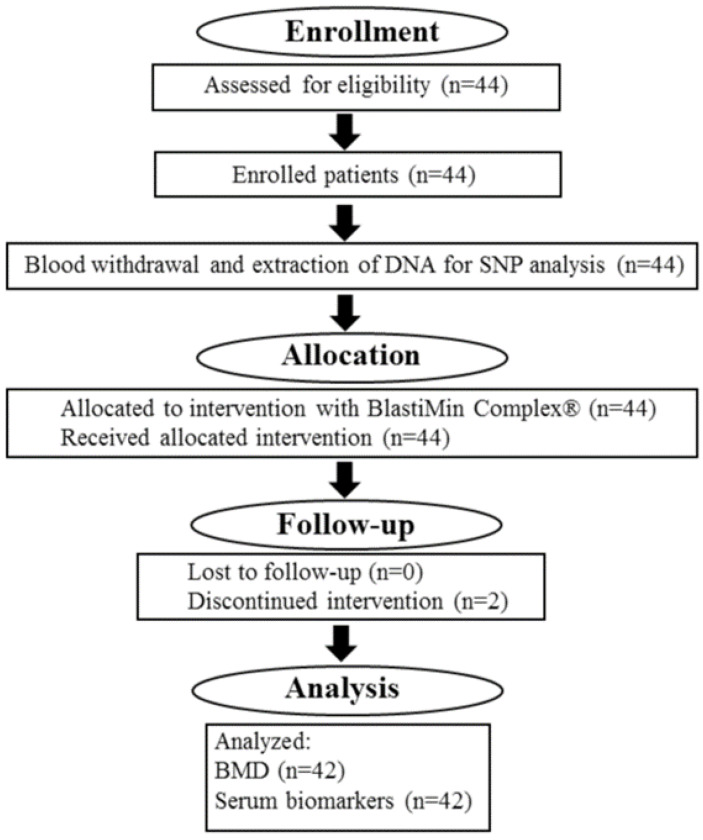
Flowchart diagram of the BlastiMin Complex^®^ clinical trial.

During the 12 months of treatment, patients took one BlastiMin Complex^®^ tablet per day 10 min before the main meal. They were administered a nutraceutical composition of 200 mg of extract of *Curcuma longa* (curcuminoids 95%), 60 mg of quercetin, 30 mg of polydatin, 25 mg of orthosilicic acid, 150 μg of vitamin K2, and 2.000 I.U. of vitamin D3. Clinical data were obtained at the baseline (t0), after 6 months of treatment (t6), and after 12 months of treatment (t12). Treatment compliance was assessed by counting the number of nutraceutical doses returned at the time of specific clinic visits. The data of patients are reported in Table 4, and the flowchart diagram of the clinical trial is described in Figure 1.

### 4.3. Assessments

All plasma parameter values were obtained after the withdrawal of the blood samples at t_0 months_, t_6 months_, and t_12 months_. Venous blood samples were centrifuged at 1300× *g* for 20 min at room temperature to obtain the separation of plasma and serum, which were divided into 500 µL aliquots and stored at −80 °C. The tubes were assigned a unique sample code, which allows for matching with clinical and laboratory databases. The following biomarkers of bone remodeling were evaluated via specific immunoassays: bALP and OCN (Liaison XL, Diasorin, Milan, Italy), CTX and P1NP (Cobas Elecsys^®^, Roche Diagnostics, Basel, Switzerland), 25-hydroxy-vitamin D2 + D3 (Atellica IM VitD, Siemens Healthineers, Erlangen, Germany), and the marker of systemic inflammation CRP (Atellica CH wrCRP, Siemens Healthineers, Erlangen, Germany).

In addition, the lumbar spine (LS) and femoral neck (FN) BMD values were analyzed at t_0 months_, t_6 months_, and t_12 months_, and the T-score values were evaluated by DXA and REMS techniques at t_0 months_ and t_12 months_. Furthermore, the genetic variants associated with increased risk of osteoporosis development were analyzed at t_0 months_, using 2 aliquots of buffy coat that were obtained from an additional blood sample withdrawn from all the enrolled patients. These samples were used to perform the genetic analyses of the selected Single Nucleotide Polymorphisms (SNPs): *CTR* rs1801197 T>C; *VDR* rs17879735 G>T; *VDR* rs1544410 G>A; *VDR* rs11568820 G>A; *VDR* rs2228570 T>C; *VDR* rs731236 T>C; *COL1A1* rs1800012 G>T; *RANK* rs3018362 A>G; *RANKL* rs9594759 C>T; *RANKL* rs9594738 C>T.

### 4.4. Polymerase Chain Reaction for the SNP Analysis

The DNA extracted from the white blood cells obtained from the blood samples of the enrolled patients has been used as the template for the multiplex-PCR experiments performed with the Labcycler instrument (SensoQuest GmbH, Göttingen, Germany), using Agena Bioscience^®^ reagents specific for the iPLEX^®^ Pro technology with MassARRAY^®^ Analyzer 4 (Agena Bioscience, San Diego, CA, USA) and a mix of primers to obtain amplified fragments, including all polymorphic sites of interest. Briefly, the reaction was set up in a 5 μL final volume using 100 ng of DNA as the template and 100 nM of each specific primer, following the manufacturer’s instructions. The sequences of the primers used for PCR assays are property of the company Diatech Pharmacogenetics S.r.l. (Jesi, Italy). For PCR amplifications, 45 PCR cycles were run with the following thermal profile: 30 s at 95 °C, 30 s at 56 °C, 60 s at 72 °C, followed by 5 min at 72 °C and then 5 min at 4 °C; before cycling, 2 min at 95 °C were allowed for DNA polymerase activation. After this amplification step, the PCR products were treated with Shrimp Alkaline Phosphatase (SAP reaction) (ThermoFisher Scientific, Waltham, MA, USA) to remove the residual nucleotides. Briefly, the reaction was set up in a 7 μL final volume using 5 μL of the PCR product and the SAP buffer, following the manufacturer’s instructions. The SAP enzymatic reaction was performed with the following thermal profile: 40 min at 37 °C, 5 min at 85 °C, and 5 min at 4 °C. Afterward, primer extension experiments were performed (iPLEX^®^ reaction) (Agena Bioscience, San Diego, CA, USA) using extension primers adjacent to every investigated polymorphic site. Briefly, the reaction was set up in a 9 μL final volume using 7 μL of SAP-treated PCR product, 70–140 nM of each specific extension primer, and the Termination Mix with the nucleotides with a modified mass. For the iPLEX PCR reaction, 40 PCR cycles were run with the following thermal profile: 5 s at 94 °C, 5 s at 52 °C, 5 s at 8 0°C, followed by 3 min at 72 °C and then 5 min at 4 °C; before cycling, 30 s at 94 °C were allowed for Thermosequenase activation (Avantor, Radnor, PA, USA). After this step, we obtained, for every individual polymorphic site, one or more analytes with a known mass, determined as the sum of the mass of the extension primer and the nucleotide with a modified mass inserted at the polymorphic site. The final analysis performed with the mass spectrometer generated a peak with known mass for every single analyte, which was associated with a wild type, mutated, or heterozygous genotype of the analyzed sample.

### 4.5. BMD Measurement

BMD was measured by dual-energy X-ray absorptiometry (DXA) at the lumbar spine (LS) (L1–L4), femoral neck, and total hip using Lunar Prodigy^®^ densitometer (QDR 4500 or Horizon or Discovery, Hologic, Marlborough, MA, USA; Lunar Prodigy, iDXA, DPX-IQ General Electric GE Healthcare, Chicago, IL, USA). The software used for the data analysis was Lunar Prodigy^®^ enCore 2007 version 11.4; BMD values were expressed as g/cm^2^. T-score was expressed as SD values and calculated according to the manufacturer’s normative data [50]. BMD data were collected at t_0 months_, t_6 months_, and t_12 months_. Furthermore, the BMD values were also evaluated using the technique of Radiofrequency Echographic Multi Spectrometry (R.E.M.S.), as previously reported [51].

### 4.6. Statistical Analysis

The data were expressed as the mean ± SD. Paired Student’s *t*-tests between the t_0 months_ data and the t_6 months_ data and between the t_0 months_ data and the t_12 months_ data were performed with Past Software version 3.20, which was used for statistical analysis of the data; differences between groups were considered statistically significant when *p* < 0.05.

## 5. Conclusions

This is the first study investigating the effects of BlastiMin Complex^®^ supplementation in women with osteoporosis or osteopenia. The treatment with this nutraceutical led to a statistically significant increase in the levels of the BTMs’ CTX and P1NP after 6 months of treatment and a further increase in the P1NP marker after 12 months of treatment, suggesting the induction of the proliferation and activity of osteoblasts. Notably, the primary endpoint of this clinical trial was the non-worsening of the T-score values, and this endpoint was successfully reached, as indicated by the clinical data obtained using the DXA and REMS techniques. Interestingly, BlastiMin Complex^®^ increased the bone turnover index of the enrolled patients after 12 months of treatment, which is of clinical significance, suggesting the generation of new bone tissue. These findings shed new light on the potential use of BlastiMin Complex^®^ in the prevention of osteoporosis condition and indicate that this nutraceutical could be used as an adjuvant in combination with synthetic drugs for an innovative therapeutic approach for osteoporosis pathology.

## 6. Patents

European Patent Application EP 4 205 733 A1.

## Figures and Tables

**Table 1:** Serum biomarkers, BMD values, and bone turnover index (BTI) at baseline and after 6 and 12 months of BlastiMin Complex^®^ treatment. The BTI was calculated as follows: P1NP values/CTX values ^1^.

Parameters	t_0 months_	t_6 months_	t_12 months_
Vitamin D2 + D3 (ng/mL)	33.62 ± 12.87	33.91 ± 9.47	32.60 ± 8.31
P1NP (μg/L)	63.64 ± 17.80	71.43 ± 26.49 *	74.30 ± 24.57 ***
CTX (pg/mL)	381.67 ± 152.14	430.88 ± 130.18 *	406.60 ± 160.42
OCN (ng/mL)	21.59 ± 5.46	20.35 ± 4.20	22.41 ± 5.92
bALP (ng/mL)	14.56 ± 6.44	14.72 ± 4.61	15.03 ± 6.02
CRP (mg/dL)	0.26 ± 0.05	0.22 ± 0.07	0.12 ± 0.09 ***
BTI	0.167	0.166	0.183
BMD L1-L4 (g/cm^2^)	0.831 ± 0.08	0.834 ± 0.07	0.829 ± 0.08
BMD FN (g/cm^2^)	0.640 ± 0.08	0.640 ± 0.07	0.627 ± 0.08

Results are presented as the mean value ± SD of the clinical data obtained from the enrolled patients. Statistical significance was evaluated using the paired Student’s *t*-test; * *p* < 0.05; *** *p* < 0.001 when compared with t_0 months_ values. ^1^ The data from 42 patients have been used for the preparation of this table.

**Table 2:** T-score values at t_0 months_ and t_12 months_, evaluated by DXA and REMS techniques ^1^.

Parameters	t_0 months_	t_12 months_
DXA L1-L4 T-score	−1.512 ± 1.029	−1.551 ± 1.014
DXA FN T-score	−1.514 ± 0.607	−1.515 ± 0.687
REMS L1-L4 T-score	−1.96 ± 0.68	−2.00 ± 0.71
REMS FN T-score	−1.88 ± 0.74	−1.98 ± 0.74

Results are presented as the mean value ± SD of the clinical data obtained from the enrolled patients. ^1^ The data from 42 patients have been used for the preparation of this table.

**Table 3:** Genotypes of the enrolled patients for the SNPs investigated in this study ^1^.

Gene	SNP	Homozygous wt	Heterozygous	Homozygous Mut
*CTR*	rs1801197 T>C	56.8%	40.9%	2.3%
*VDR*	rs17879735 G>T	29.6%	45.4%	25.0%
*VDR*	rs1544410 G>A	43.2%	43.2%	13.6%
*VDR*	rs11568820 G>A	50.0%	43.2%	6.8%
*VDR*	rs2228570 T>C	50.0%	40.9%	9.1%
*VDR*	rs731236 T>C	59.1%	47.7%	11.4%
*COL1A1*	rs1800012 G>T	68.2%	27.3%	4.5%
*RANK*	rs3018362 A>G	65.9%	59.1%	6.8%
*RANKL*	rs9594759 C>T	18.2%	54.5%	27.3%
*RANKL*	rs9594738 C>T	22.7%	59.1%	18.2%

^1^ The data from 44 patients have been used for the preparation of this table.

**Table 4:** Patient demographic data ^1,2^.

Characteristics	
Sex, no. (%)	
Male	0%
Female	100%
Age, y	62.4 ± 6.1
Weight, kg	63.5 ± 9.8
BMI	24.1 ± 3.5

^1^ The data from 44 patients have been used for the preparation of this table. ^2^ None of the patients were on any other medication for osteoporosis apart from BlastiMin Complex^®^.

## Data Availability

The data presented in this study are available upon request from the corresponding author. The data are not publicly available due to privacy.
